# The impacts of acid suppression on duodenal microbiota during the early phase of severe acute pancreatitis

**DOI:** 10.1038/s41598-020-77245-1

**Published:** 2020-11-18

**Authors:** Xiao Ma, Libin Huang, Zhiyin Huang, Jinsun Jiang, Chong Zhao, Huan Tong, Zhe Feng, Jinhang Gao, Rui Liu, Mingguang Zhang, Ming Zhou, Qinghua Tan, Ling Liu, Chengwei Tang

**Affiliations:** 1 Laboratory of Gastroenterology and Hepatology, State Key Laboratory of Biotherapy, West China Hospital, Sichuan University, Chengdu, People’s Republic of China; 2 Department of Gastroenterology, West China Hospital, Sichuan University, Guoxue Alley 37#, Chengdu, 610041 People’s Republic of China; 3 Department of Clinical Research Management Clerk, West China Hospital, Sichuan University, Guoxue Alley 37#, Chengdu, 610041 People’s Republic of China

**Keywords:** Pancreatic disease, Dysbiosis

## Abstract

Duodenal dysbiosis may be potential infection risks in patients with severe acute pancreatitis (SAP). Acid-suppression drugs (ACDs) are widely used in SAP patients in Asian countries. However, the impact of ACDs on duodenal microbiota during the early phase of SAP is still unknown. This randomized controlled clinical trial evaluated the impacts of esomeprazole (Eso), one of ACDs on duodenal microbiota during the first week of SAP with duodenal aspirates culture and 16sRNA Illumina sequencing analysis. 66 patients were randomized as 1:1 ratio into Eso group (Eso 40 mg/day) and Eso-N group (no Eso). The occurrence of duodenal bacterial overgrowth (DBO) was significantly higher in Eso group (about 85%) than that in Eso-N group (about 42%). The duodenal microbiota of the SAP patients shifted away from that of the normal control. There were differences between the Eso-N and Eso groups including enriched abundances of the class Negativicutes, order Selenomonadales and genus *Veillonella*. Acid suppression significantly increased incidence of Candida oesophagitis (CE) by 8-folds but did not increase other infectious events. In conclusion, acid suppression greatly increased the occurrence of DBO, duodenal dysbiosis and CE during the first week of SAP. Restrictive use of acid-suppressing medications might be helpful to reduce CE and potential risk of pancreatic infection in SAP patients.

**Trial registration**: Chictr.org, ChiCTR-IPR-16008301, Registered April 18 2016, http://www.chictr.org.cn/showproj.aspx?proj=14089.

## Introduction

Severe acute pancreatitis (SAP) is one of the leading causes of hospital admissions for gastrointestinal disorders with a high mortality rate. Despite of some progress, SAP remains a serious challenge^1^. The strategy of “putting pancreas at rest” by inhibiting of pancreatic secretion is still taken as the conventional treatment for acute pancreatitis (AP)^2^. Physiologically, the reduction of gastric acid secretion may help to inhibit pancreatic secretion. Therefore, proton pump inhibitors (PPIs), one of the most effective acid-suppression drugs (ASDs), are widely prescribed for AP in Asian countries. PPIs are recommended for AP treatment in Chinese guidelines in 2019^3^. The data from a Japanese national administrative database showed that PPIs were used in approximately 37.3% (3879/10,400 cases) of SAP patients^4^. A similar situation also exists in Korea^5^. However, there is less evidence for the benefit of PPI use in SAP.

The gastric acid barrier has been assumed to be crucial to maintain homeostasis of the gastrointestinal microbiota^6^. Acid suppression may alter the intra luminal environment of the upper gastrointestinal tract and alter the gut microbiome. Several studies have reported that PPIs increase the risk of small intestinal bacteria overgrowth (SIBO) and alter the composition of gut microbiota^7–9^. Intestinal dysbiosis and involvement of the innate immune system may culminate the inflammatory cascade. Therefore, gut has been taken an inflammatory amplifier and plays an important role during the development of SAP. Accumulated experimental studies have shown an association between intestinal bacterial overgrowth and the severity of AP^10,11^. It is worth noting that there is a positive correlation between duodenal bacteria overgrowth (DBO) and bacterial infections in experimental AP^12^. Normally, few bacteria are detected in the duodenum, but PPI-induced hypochlorhydria may result in DBO^13^. Moreover, the application of ASDs might increase the occurrence of *Candida* oesophagitis (CE)^14^. Nevertheless, duodenal dysbiosis and ASDs-associated CE in SAP patients are still largely unknown.

This study was aimed to assess the impacts of acid suppression on the duodenal microbiota and other potential infection risks in SAP patients during the early phase of disease.

## Results

### Patient characteristics and recruitment flow

**Figure 1:**
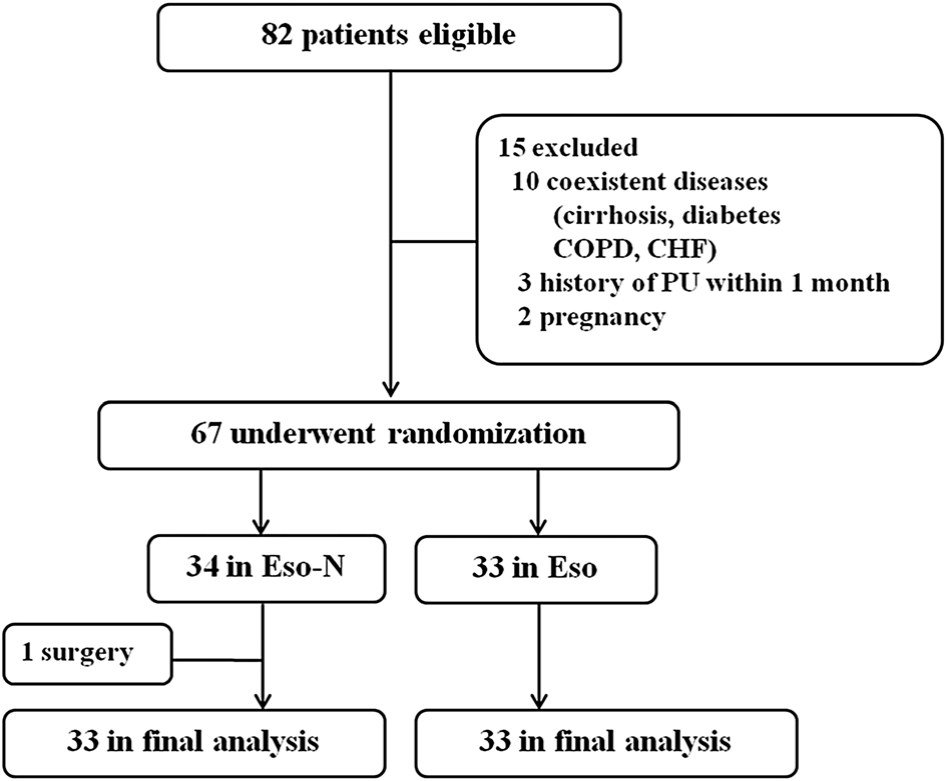
Flow diagram of the trial. *COPD* chronic obstructive pulmonary disease, *CHF* chronic heart failure, *PU* peptic ulcer.

From April 2016 to March 2017, 67 patients were directly admitted to our hospital and were enrolled randomly into the Eso group (conventional treatment plus esomeprazole, 40 mg/day; n = 33) and the Eso-N (conventional treatment, n = 34) groups. One patient was lost from the Eso-N group due to splenic artery haemorrhage and was transferred to surgery. A total of 33 patients were included in each group finally (Fig. 1). The baseline data for the two groups were comparable (Table 1). Seven of 66 patients accepted antibiotics as indicated biliary infection or massive necrosis during the early phase of SAP. The distribution of those cases between the two groups was similar.

**Table 1:** Characteristics of the patients at baseline.

	Eso-N (n = 33)	Eso (n = 33)	*p* value
Age, year	44.55 ± 9.29	46.12 ± 11.14	0.535
Female, n (%)	13 (50)	10 (37)	0.438
BMI, kg m^−2^	25.50 ± 2.34	26.09 ± 3.62	0.438
APACHEII score	9.76 ± 4.03	9.24 ± 2.49	0.535
Marshall scores	2.38 ± 1.12	2.21 ± 1.02	0.591
**Organs involved, n (%)**			
Respiratory	27 (81.81%)	25 (75.75%)	0.382
Renal	5 (15.15%)	7 (21.21%)	0.375
Cardiovascular	1 (3.03%)	2 (6.06%)	0.5
CRP , mg/ml	249.93 ± 114.9	254.82 ± 132.79	0.873
Feeding tube, n (%)	11 (33.3)	8 (24.2)	0.294
**Etiology, n (%)**			0.446
Biliary	20 (60.6)	15 (45.5)	
Alcohol	2 (6.1)	4 (12.1)	
Others	2 (6.1)	5 (15.2)	
Uncertain	9 (27.3)	9 (27.3)	
Antibiotics in early phase, n (%)	4 (12)	3 (9)	0.689
Initiation of enteral nutrition, day	3.4 ± 1.6	3.8 ± 1.8	0.344
Mortality	0	0	–

*BMI* body mass index, *APACHE II* acute physiology and chronic health evaluation, *CRP* C-reaction protein.

### More DBO was found in SAP patients with hypochlorhydria

**Figure 2:**
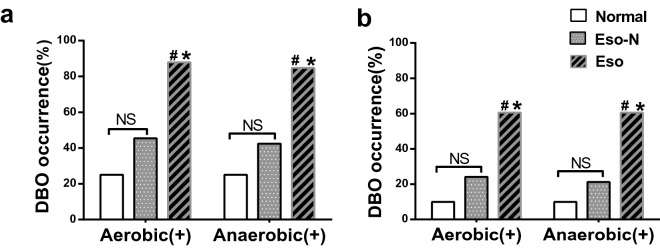
Occurrences of DBO in the three groups. (**a**) Bacterial growth > 10^3^ CFU/mL. (**b**) Bacterial growth > 10^5^ CFU/mL ^#^*p* < 0.05 versus Eso-N group; *p < 0.05 versus volunteer group; ^NS^*p* > 0.05 versus normal control group; *DBO* duodenal bacteria overgrowth.

The intra-gastric pH of the Eso group was significantly higher than that of the Eso-N group (5.15 ± 1.46 vs. 2.70 ± 0.93, *p* < 0.001). The intra-gastric pH of the normal control group (n = 20) was 2.16 ± 0.70. The bacterial cultures from the endoscopic channel were negative. The occurrence rates of DBO (bacterial concentration > 10^3^ CFU/mL) were highest in the Eso group among three groups (Fig. 2a) either in aerobic culture (87.9%, 45.4% and 25%) or in anaerobic culture (84.8%, 42.4% and 25%), *p* < 0.001. The results were similar even when DBO was defined as a bacterial concentration > 10^5^ CFU/mL (Fig. 2b).

### Characteristics of the duodenal microbiota shift in SAP patients with hypochlorhydria

Sixty patients were eventually included in the microbiota analysis (30 in Eso group, 30 in Eso-N group). Illumina paired-end sequencing of duodenum mucosa generated an average of 31,989 ± 18,006 clean tags and a total of 11,022 OTUs. At an even length of 10,000 sequences per sample, OTUs at 97% similarity threshold for mucosa microbiota were clustered.

**Figure 3:**
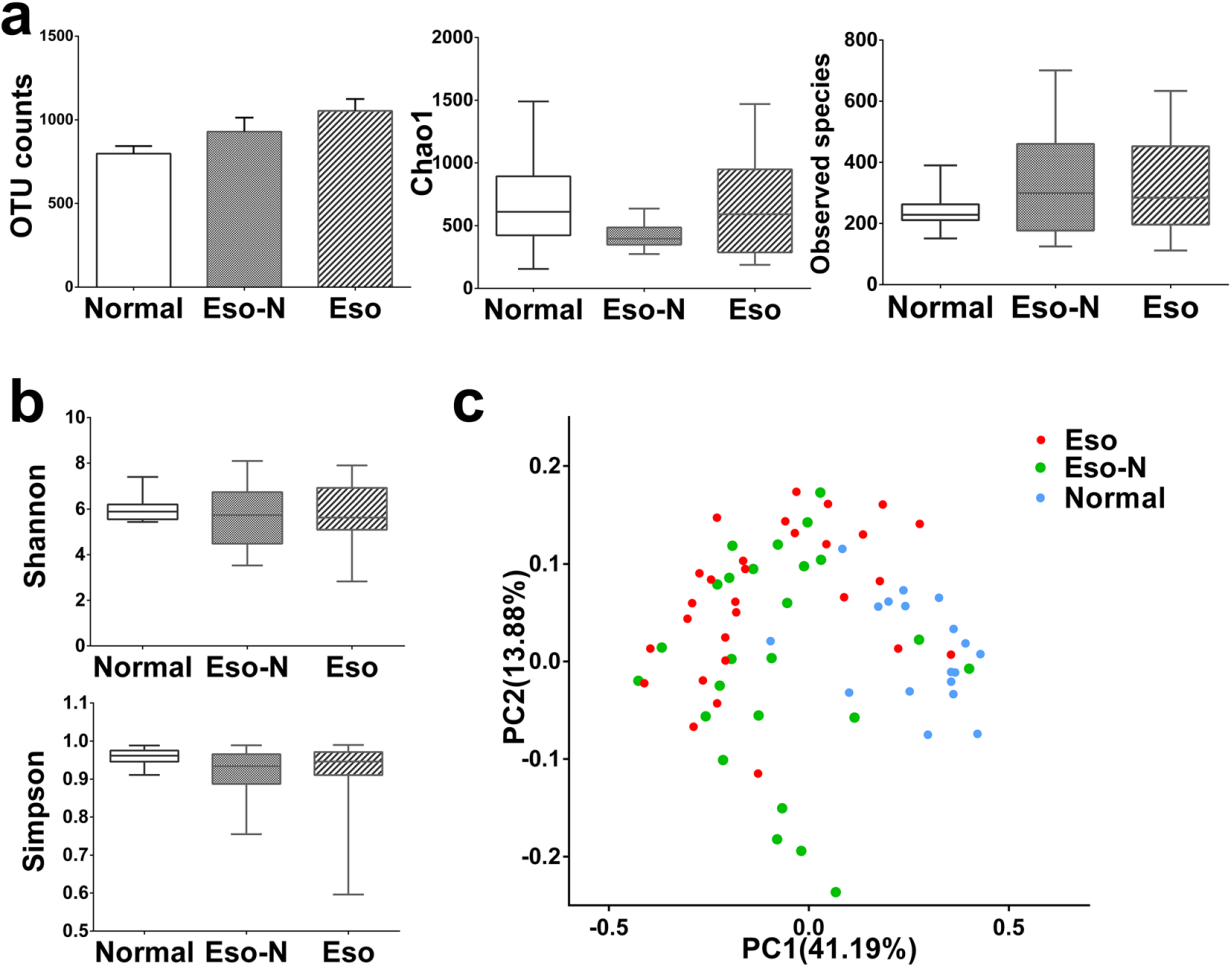
Comparison of α and β diversity in the three groups. (**a**) No richness significant differences between Eso and Eso-N group (ANOVA analysis). (**b**) No α diversity significant differences between Eso and Eso-N group (ANOVA analysis). (**c**) Principal coordinate analysis (Weighted unifrac index) of all samples at the OTU level revealed distinct clustering of duodenum mucosa microbiota.

**Figure 4:**
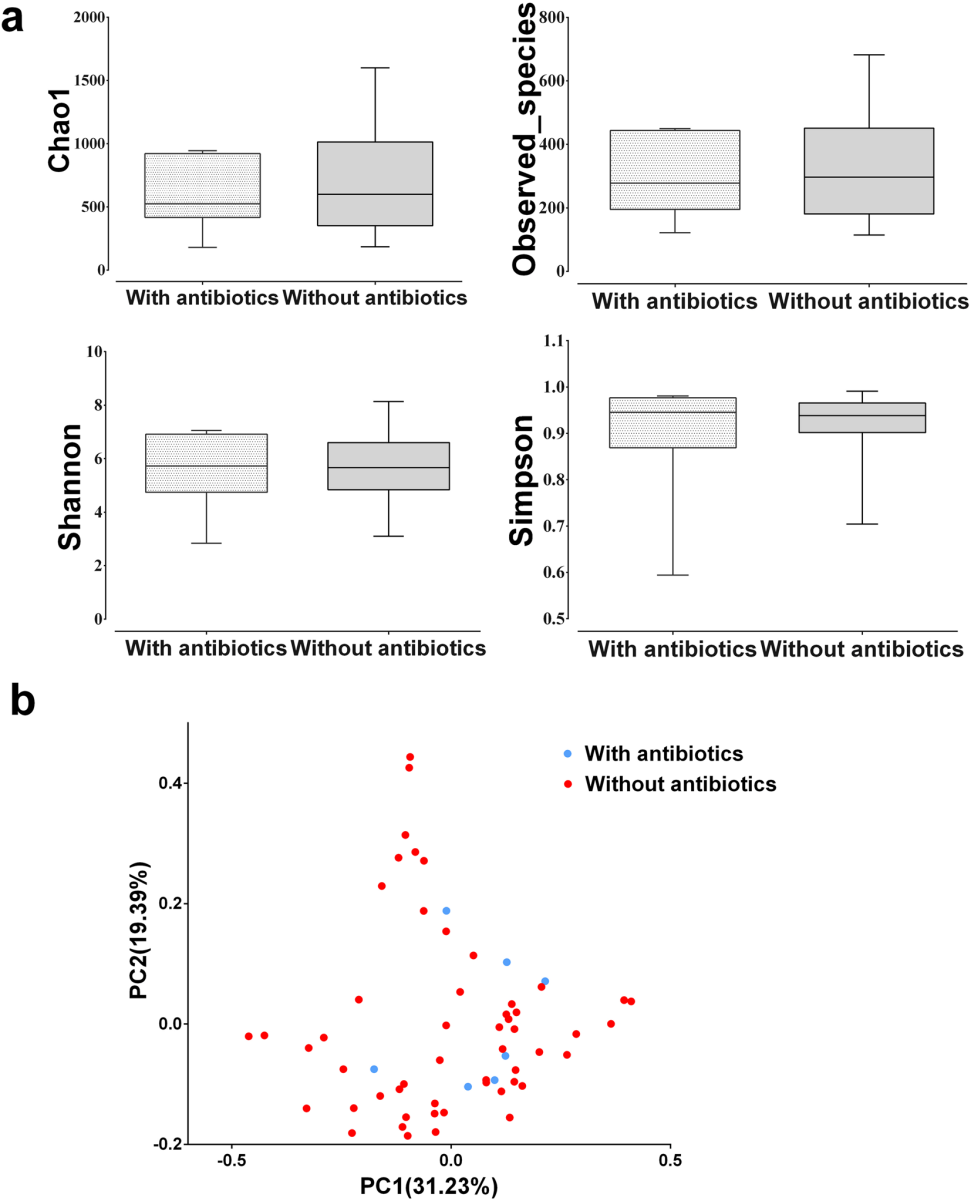
The changes of duodenal microbiota in patients with or without antibiotics treatments.

No significant difference was found between the Eso and the Eso-N group in the α-diversity of duodenal microbiota, richness as determined by OTUs, Chao1 index or observed species (Fig. 3a) and diversity as determined by the Shannon or Simpson diversity indices (Fig. 3b). Principal co-ordinate analysis (Weighted UniFrac index) of all samples at the OTU level revealed clustering of duodenal microbiota in SAP patients that was distinct from the normal control for PC1, *p* = 0.026 (Fig. 3c). Compared with the Eso group of SAP patients, the composition of duodenal microbiota in the Eso-N group was heterogeneous for PC2, (Fig. 3c). The heat-map further demonstrated the difference in duodenal microbiota between Eso-N and Eso group (Supplementary Figure S1). In total, 36 taxa were significantly different between Eso-N and Eso group. The relative abundances of representative taxonomies p_ Firmicutes, c_ Negativicutes,c_ Gammaproteobacteria, o_Selenomonadales and o_Enterobacteriales were significantly enriched in the Eso group compared with the Eso-N group. There was no significant difference in α-diversity or β-diversity between the patients treated with (n = 7) or without (n = 53) antibiotics. Principal co-ordinate analysis also showed no distinct clustering of duodenal microbiota between the treatment with or without antibiotics (Fig. 4).

**Figure 5:**
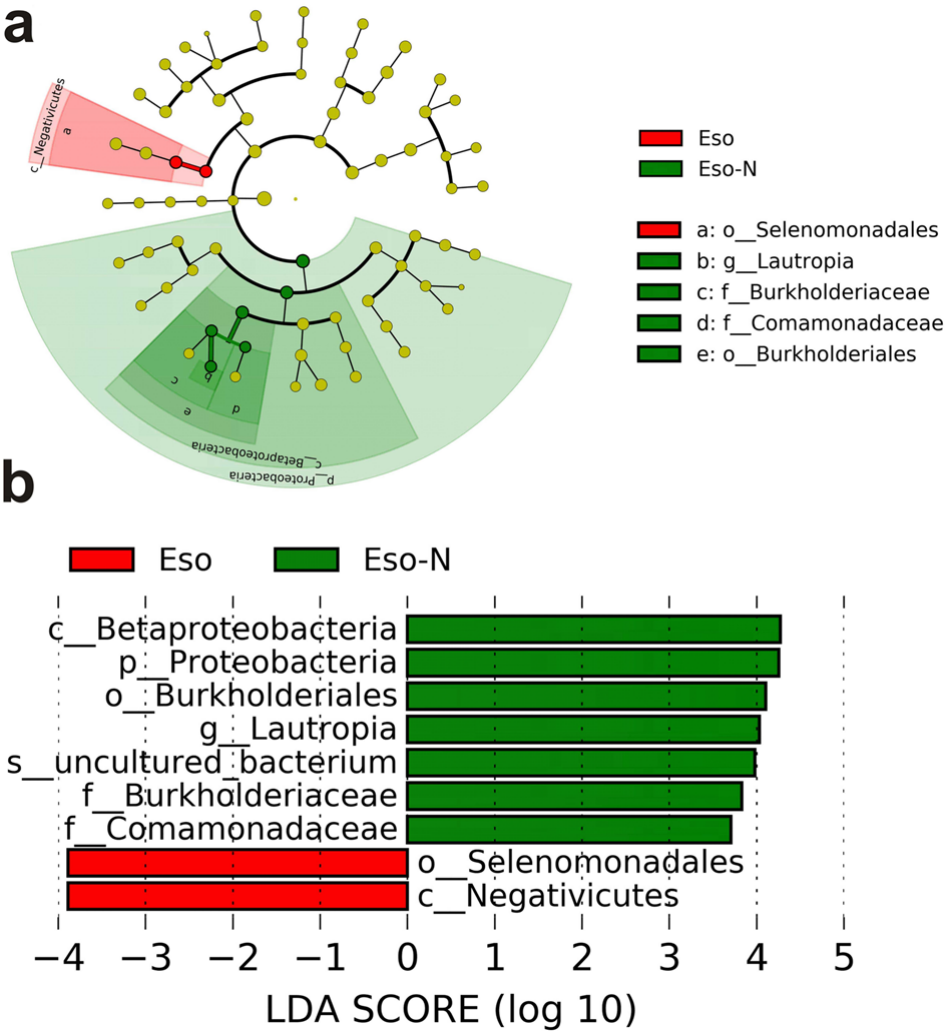
Microbiota changes in duodenum of two groups. (**a**) Linear discriminant analysis effect size (LEfSe) analysis of enriched bacterial taxa. The colored nodes from the inner to the outer circles represent the abundant taxa from the phylum to the genus level. The red labels represent the taxa enriched in the Eso group while the green labels represent the taxa enriched in the Eso-N group; (**b**) linear discriminant analysis (LDA) revealed the effect size of each differentially featured taxa(LDA > 2, *p* < 0.05).

**Figure 6:**
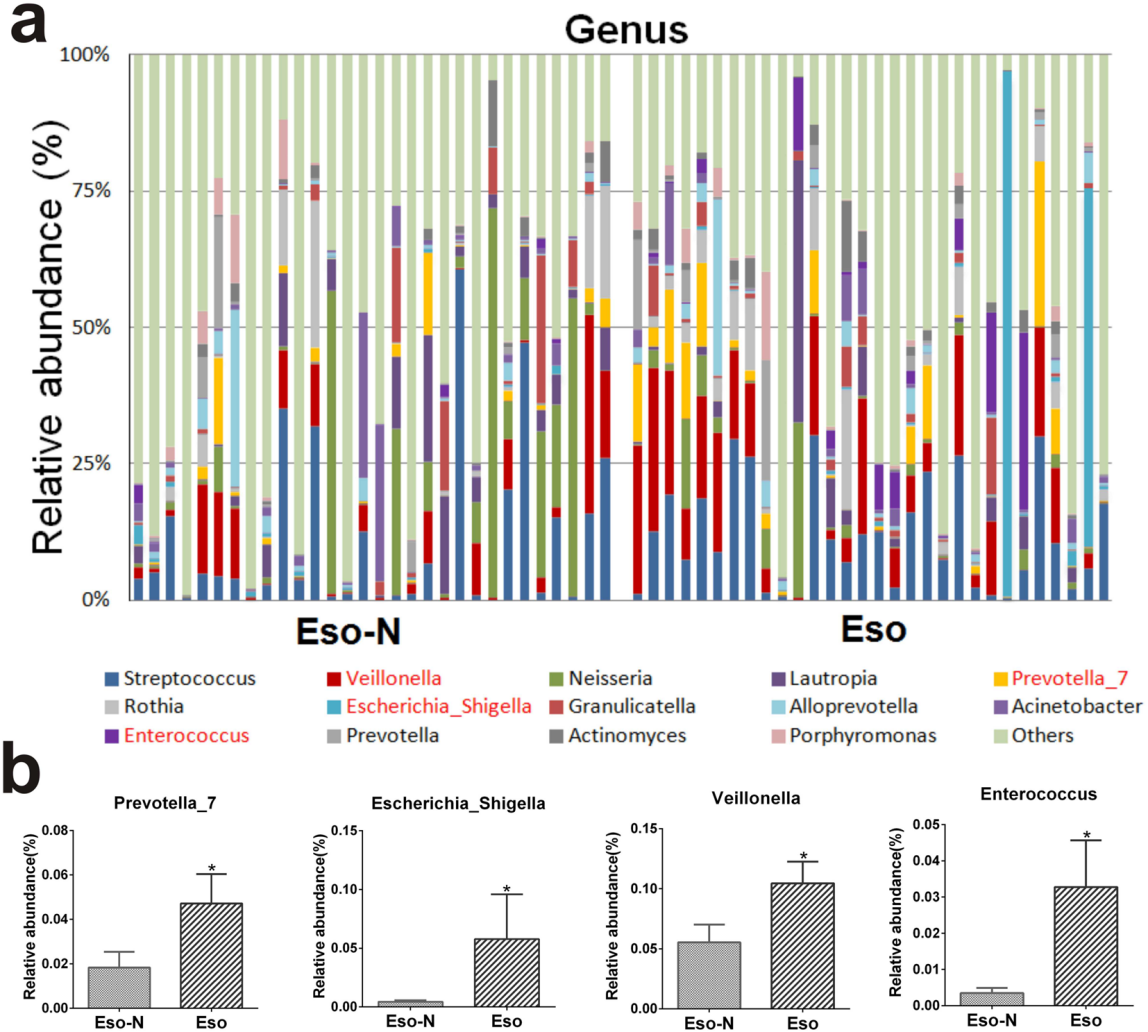
Taxonomic compositions of samples at genus level in two groups. (**a**) Taxonomic composition. (**b**) Percentage abundance of increased taxonomies at genus level: taxons with red color in figure (**a**), *FDR-*p* < 0.05.

The over-representation of bacteria at different taxonomic levels in response to esomeprazole therapy was characterized using a LEfSe analysis and LDA methods (Fig. 5). There were profound changes in duodenal microbiota between the two groups. P_ Firmicutes, c_ Negativicutes, o_ Selenomonadales were considerably enriched in patients on the esomeprazole treatment. The relative abundances of dominant bacteria at the genus level were shown in Fig. 6a. At the genus level, the enrichment of *Enterococcus*, *Veillonella*, *Escherichia_Shigella* and *Prevotella_7* (Fig. 6b) was associated with esomeprazole treatment. Combined with the previous LDA results (Fig. 5b), the genus *Veillonella* in the order Selenomonadales was increased in patients with esomeprazole use. The genus *Streptococcus*, which is regarded as a key indicator of PPI-induced changes in upper gastrointestinal tract microbiota, was higher in the Eso group but not significantly different from the Eso-N group (11.53% *vs.* 10.67%, adjusted *p* = 0.052).

### Higher occurrence of *Candida* oesophagitis in SAP patients with hypochlorhydria

The occurrence of CE in SAP patients was significantly higher in the Eso group than in the Eso-N group (24.2% *vs.* 3.0%, *p* = 0.013). Based on Kodsi grading, all cases of CE were mild (grade I/II). All patients with CE in this study were asymptomatic. Duodenogastric reflux was detected in 48.5% (32/66) of patients in this study and there was no significant difference between the two groups (45.5% *vs.* 51.5%, *p* > 0.05). More co-existing infection events were found in the patients with CE than in those without CE (77.8% *vs.* 36.8%, *p* = 0.026). Other extra-pancreatic infections in the Eso group did not differ from the Eso-N group (Table 2). In the Eso group, a patient with a persistent fever of approximately 38 °C and massive necrosis was suspected to suffer pancreatic infection during the first week of the disease. Although antibiotics had been prophylactically prescribed during the early stage of his illness, pancreatic infection was not avoided at the later stage after the onset of pancreatitis.

**Table 2:** Infections events in two groups.

Infections, n (%)	Eso-N (n = 33)	Eso (n = 33)	*p* value
Candida esophagitis	1 (3)	8 (24.2)	0.013
Pneumonia	10 (30.3)	13 (39.4)	0.303
Bacteremia	1 (3)	3 (9.1)	0.307
Urinary tract	1 (3)	2 (6.1)	0.5
Suspicious infected pancreatic necrosis	0	1 (3)	0.5

### Impacts of hypochlorhydria on clinical scores of SAP patients

There were no significant differences in the APACHE II, SIRS or Marshall scores between the Eso-N and Eso groups at day 4 and day 7 of treatment, *p* > 0.05 (Supplementary Table S1).

### Safety

No patients suffered GI bleeding after specimen collection procedure. All of the patients finished the endoscopy safely without any exacerbation of respiratory failure during or after the gastroscopy in this study.

## Discussion

SAP, which is characterized by the inflammatory cascade, often results in gut bacterial overgrowth, which in turn aggravates the disease through activation of the innate immune system or bacterial translocation ^15–17^. Bacteria derived from the small bowel rather than from colon may be more important in the development of SAP^11,12^. Compared with the glucose breath test, duodenal or jejunal aspirate culture is considered a more accurate test to detect small intestinal bacteria overgrowth (SIBO)^18^. Using duodenal aspirate culture, this study showed that SAP increased the risk of duodenal bacteria overgrowth (DBO) regardless of whether DBO was defined as 10^3^ CFU/mL or 10^5^ CFU/mL^19^. Both aerobic and anaerobic were overgrown in the duodenum of SAP patients. In addition, the composition of duodenal microbiota was shifted away from the normal control by SAP.

In the context of SAP, a short-term application of esomeprazole doubled the occurrence of DBO due to higher intragastric pH. The composition of duodenal microbiota in SAP patients without acid suppression was heterogeneous and lack of a dominant bacterial cluster. With the acid-suppression treatment, the duodenal microbiota of SAP patients became less heterogeneous and formed some dominant bacterial clusters. The relative abundance of the class *Negativicutes*, order *Selenomonadales* and genus *Veillonella* may form the dominant bacterial clusters; their implication for SAP clinical course requires further investigation. Some pathogenic bacteria such as g_*Enterococcus*, g_*Veillonella*, g_*Escherichia_Shigella*, g_*Prevotella* were greatly increased after acid suppression and are considered indicative of susceptibility to enteric infections^20–22^. Although there were seven cases ever accepted antibiotics during esomeprazole use in this study, the α-diversity and β-diversity of their microbiota did not show significant differences compared with those of the 53 patients who did not take antibiotics. A potential difference may be masked by the small sample of cases (n = 7) with antibiotics. The impact of approximately 10 percent on antibiotics in each group did not affect the significant role of acid suppression on DBO or dysbiosis.

It has been reported that SIBO/DBO was positively correlated with the severity of experimental pancreatitis^12^. However, duodenal bacterial overgrowth and dysbiosis secondary to acid suppression did not show an impact on the clinical scores of SAP patients suggesting that the organ failure score may be affected directly by the inflammatory cascade instead of DBO. In this study, the treatment of acid-suppression did not increase infection events except the CE. Pneumonia was the most common infections in this study, which might due to the higher incidence of acute lung injury or acute respiratory distress syndrome (75–82%) in the study and the treatment of mechanical ventilation. Similar results also showed that PPI inhibitors were not associated with an increased risk of bloodstream infections in the intensive care unit^23^. Although the actual clinical problems were not serious, the theoretical risk of SIBO/DBO or SAP aggravation due to duodenal dysbiosis remains not to be ignored. Additional well-designed and sophisticated clinical studies are necessary.

CE is one of the most common infections of the esophagus and is caused by the yeast *Candida*. The prevalence of CE was approximately 3.8% in an endoscopic study containing 1855 subjects^24^. PPIs or histamine-2 blockers were considered the major risk factors of CE in HIV-negative patients^25^. Several studies have reported that 4- week acid suppression treatment may greatly increase CE in HIV-negative patients^26^. Although fungal infections are often detected in patients with SAP^27^, there is less data on the occurrence of CE in AP. Surprisingly, approximately 13.6% (9/66) occurrence of CE was found in SAP patients in this study. SAP patients with acid suppression showed an approximately eightfold higher occurrence (24.2%) of CE than patients without acid suppression (3%). Moreover, it only took 1 week to significantly increase the risk of CE in SAP patients as a result of acid suppression, whereas it took 4 weeks to develop CE in HIV-negative patients^26^. Fortunately, all cases of CE in this study were mild. In addition, patients with CE suffered more coexisting infections than patients without CE. It is not clear whether CE would be a precursor of fungal infection in a necrotic pancreas. In this study, only 1 patient suffered suspicious pancreatic infection. The low incidence of suspicious pancreatic infection might due to the observation time point of this study. Infected necrosis usually occurred during the later phase (4–8 weeks after onset) of SAP, but most of the SAP patients in this study were at the early stage (< 2 weeks) of SAP. Fungi and bacteria sharing the same host have some competitive interactions with each other. Duodenal dysbiosis may have a potential influence on upper gastrointestinal tract fungi (the gut 'mycobiome') or may in turn affect disease courses^28^. Obviously, SAP and DBO may accelerate the occurrence of CE secondary to acid suppression in a short time.

There are several limitations to this study. It is a single-centre study, which could bias the data. A well designed, multiple-centre study would be more convincing. This study was focused on the effect of acid suppression on the duodenal microbiota during the early phase of SAP. The follow-up period was designed as 1 week, because the primary outcome of the study was a significant difference in the positive rate of DBO between the two groups. The complications in the later phase of SAP are affected by many risk factors and DBO may be one of them. The aim of this pioneer study was to firstly confirm that DBO may be increased by ACDs. However, the potential clinical complication on SAP in the later phase is also important. The next trial may investigate the effects of multiple factors including DBO increased by acid-suppression drugs on infected necrosis of pancreas in SAP patients. This study ignored duodenal specimen sequencing with internal transcribed spacer identification. So that, the composition and diversity of fungal species in duodenum is not clear. The crosstalk between CE and duodenal dysbiosis in SAP patients should be addressed.

## Conclusion

Acid suppression in SAP patients greatly increased the occurrences of DBO, duodenal dysbiosis and CE in the first week of the disease. Restrictive use of acid-suppressing medications might be helpful to reduce CE and potential risk of pancreatic infection in SAP patients.

## Methods

### Study design and subjects

This parallel randomized single-centre study was designed and conducted in the gastroenterology department of West China Hospital, from April 2016 to March 2017. The protocol was approved by the Chinese Ethics Committee for Registering Clinical Trials (ChiECRCT-20160018) and all research was performed in accordance with the relevant guidelines. This study adheres to the CONSORT guidelines and all authors had access to the study data and reviewed and approved the final manuscript.

Written informed consent was obtained from each subject prior to enrollment. Male and female patients aged 18–65 years were enrolled within 72 h after the onset of abdominal pain. AP was defined according to the 2012 Atlanta criteria^29^. SAP was defined as persistent organ failure (> 48 h) in patients with AP. The presence of organ failure is defined according to the modified Marshall score as a score of 2 in at least one of the three organ systems (respiratory, renal and cardiovascular system)^29^. The acute physiology and chronic health evaluation II (APACHE-II) score and modified Marshall score of each patient were evaluated when they were enrolled. The pathogens of SAP were recorded. Twenty healthy volunteers aged 18–65 years who undertook a routine health check were recruited as a normal control for the measurement of duodenal microbiota. The following patients with SAP were excluded under the following criteria: abdominal compartment syndrome; utilization of antibiotics or PPIs within 1 month; regular medical treatments with non-steroidal anti-inflammatory, antidepressant or immune suppression drugs; comorbidities including diarrhoea, chronic constipation, peptic ulcers, inflammatory bowel disease, diabetes, cirrhosis, cardiac dysfunction, chronic obstructive pulmonary disease, chronic renal insufficiency, and malignancies; drug abuse or psychosis; past history of gastrointestinal tract surgery; and pregnant or lactating women.

### Randomization and intervention

Eligible SAP patients were included and assigned to either the Eso group (conventional treatment plus esomeprazole, 40 mg/day) or the Eso-N group (conventional treatment) using computer-generated random numbers. A nurse who was not directly involved in medical care was assigned to allocate the eligible SAP patients into two groups with the sequence number concealed in an envelope. Blind group assignments were maintained for investigators involved in data collection and endoscopic procedure, as well as the technicians who cultured the bacteria or performed the 16S rRNA Illumina sequencing analysis until all data collection and data queries had been completed and the database was locked.

All clinical data were evaluated through recorded histories taking and clinical laboratory tests. All enrolled patients received conventional management according to international AP guidelines and guidelines from the Chinese association of pancreatology^3,30^. Intravenous esomeprazole (Nexium^®^, AstraZeneca, London; 40 mg) was administered to the Eso group once a day from the first day to the seventh day of enrolment.

### Sample collection using upper gastrointestinal tract endoscopy

All patients underwent upper gastrointestinal tract endoscopy (Olympus GIF-260, Japan) on the seventh day after enrolment. The tolerance of endoscopy for the enrolled patients was well assessed before the procedure by a multi-disciplinary team including experienced gastroenterologists, anesthesiologists and endoscopists. Furthermore, the gastroscopy was performed by designated experienced endoscopists to ensure accurate examination as soon as possible. An anesthesiologist monitored the patient's condition during gastroscopy. The duodenal aspirate was collected from the third segment of the duodenum with a sterile catheter through the channel of the endoscope^18^. The single-use sterile cytological brushes (Micro-Tech^®^, Nanjing, China) which pass through the endoscopic channel without collecting the mucosa, were also cultured as the blank control. No suction was performed prior to the duodenal aspirate. The aspirates were maintained in a sterile container for bacterial culture. The sterile cytological brushes were used to collect duodenal epithelium and mucus at the same location for the 16 s rRNA gene sequencing analyses. Gastric juice was suctioned from the fundus for the pH test. Suspicious lesions of oesophageal *Candidiasis* were brushed and identified under a microscope (Supplementary Figure S2).

### Colony-forming culture and 16S rRNA Illumina sequencing

A 0.5–1 mL duodenal aspiration was immediately sent to the microbiology laboratory in our hospital for aerobic and anaerobic culture. Incubation of anaerobic and aerobic bacteria was performed on blood agar and incubated anaerobically or aerobically at 37 °C for 48 h. The bacterial counts were summarized as colony forming units (CFUs) per sample. Bacterial growth > 10^3^ CFU/mL was considered as DBO^19^. Duodenal brush samples were frozen at − 150 °C until further processing. The total bacterial genomic DNA was extracted from the mucosal content and the V4-V5 variable regions of the 16S rRNA genes were amplified by PCR using universal primers 515F (-5′-GTGCCAGCMGCCGCGG-3′) and 907R (- 5′-CCGTCAATTCMTTTRAGTTT-3′). The amplicon quality was verified by gel electrophoresis and the final amplicons were quantified using a Qubit dsDNA assay kit (Invitrogen™, California, USA). Equal amounts of purified amplicon were pooled for subsequent sequencing. The amplicons were sequenced using an Illumina Miseq platform^31^. Bioinformatics analyses were performed as described previously^32^; raw sequencing data were in FASTQ format. Sequence reads were aligned using QIIME (v1.8.0)^33^ and clustering to generate operational taxonomic units (OTUs) at 97% sequence similarity. OTUs were clustered and classified using Silva database or Greengenes database (16S)^34^ and then analyzed using QIIME software for taxonomy comparisons between biological samples. The α- and β-diversity indices were calculated using QIIME. Diversity and richness plots were generated in GraphPad Prism (V.6.0). Beta-diversity was measured by calculating phylogenetically based binary Jaccard distances. Principal coordinate analysis (PCoA) was applied to the resulting distance matrices to generate plots using the default settings of PRIMER-6^35^.

### The clinical outcomes

A significant difference in DBO positive rates between groups was defined as the primary outcome. The secondary outcomes included intra-gastric pH, any infection events, microbiota shifts and the clinical scores including the APACHE II, systemic inflammatory response syndrome (SIRS) and the Marshall scores on the fourth and seventh days after admission. Any infection events were documented.

### Sample size estimation and statistical analysis

The sample size calculation was based on previous trials^7^ and our pilot study for a median overall 35% decrease in DBO incidence without PPI treatment. A sample size of 33 cases in each group would provide a 15% drop-out rate and 90% power to estimate the overall incidence decrease at the 5% level of significance in this study.

Between-group differences were evaluated using the Student’s t test for continuous variables and Pearson’s χ^2^ test for categorical variables. A one-way ANOVA was used to compare DBO incidences among the three groups. To analyse the microbiota sequences, a Kruskall–Wallis or Mann–Whitney test was used to compare the OTUs and taxonomy abundances. The resultant *p-*values were FDR (false discovery rate) corrected using the q-value package in R with a significance threshold of 5%. The heatmaps were generated using the pheatmap R package. The LDA (linear discriminant analysis) and LEfSe (linear discriminant analysis effect size) method^36^ were utilized to compare and visualize significant differences in taxa between groups.

### Ethics approval and consent to participate

The trial was approved by Chinese Ethics Committee of Registering Clinical Trials (ChiCTR-IPR-16008301). Written informed consent was obtained from each subject in this study.

## Supplementary information

Supplementary Information

## Data Availability

All data generated or analyzed during this study are included in this published article (and its Supplementary Information files).
