# Supplementary material for: The impacts of acid suppression on duodenal microbiota during the early phase of severe acute pancreatitis
Source: Sci Rep. 2020 Nov 18;10:20063. doi: 10.1038/s41598-020-77245-1 (PMC7674417; doi:10.1038/s41598-020-77245-1)
Supplement: Supplementary file 1 — Supplementary Information [file 41598_2020_77245_MOESM1_ESM.pdf]

The impacts of acid suppression on duodenal microbiota during the early phase of severe acute pancreatitis

Xiao Ma, Libin Huang, Zhiyin Huang, Jinsun Jiang, Chong Zhao, Huan Tong, Zhe Feng, Jinhang Gao, Rui Liu, Mingguang Zhang, Ming Zhou, Qinghua Tan, Ling Liu*, Chengwei Tang*

Corresponding author: Chengwei Tang (shcqcdmed@163.com) and Ling Liu (lingzipurple@163.com)

Supplementary Figure S1 Heat-map of significantly altered bacterial taxa in two groups.

Supplementary Figure S2 *Candida* oesophagitis detected by endoscopy in this study

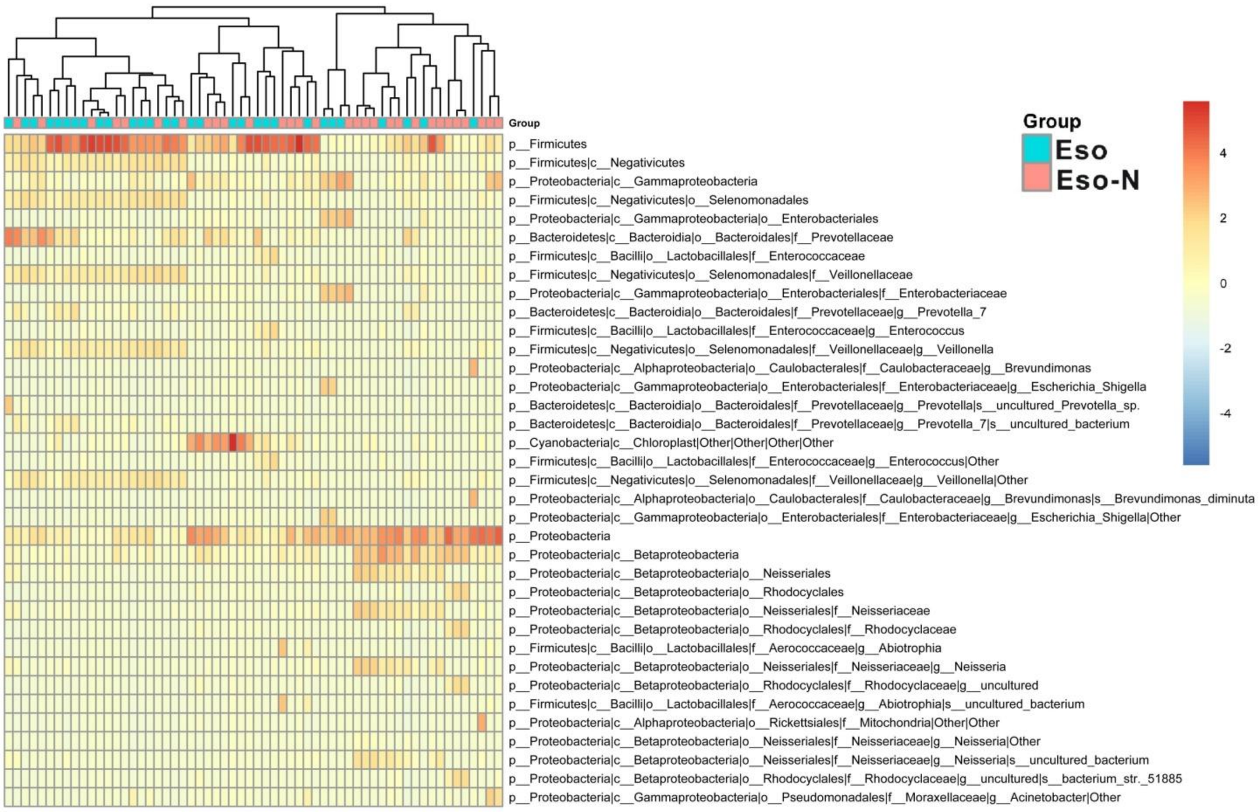

Supplementary Figure S1 Heatmap of significantly altered bacterial taxa in two groups. This heat-map depicts relative abundance of significantly altered bacteria taxa at different levels (phylum, class, order, family, genus and species) in Eso and Eso-N group, with significance (FDR multiple comparison adjusted $p<0.05$).

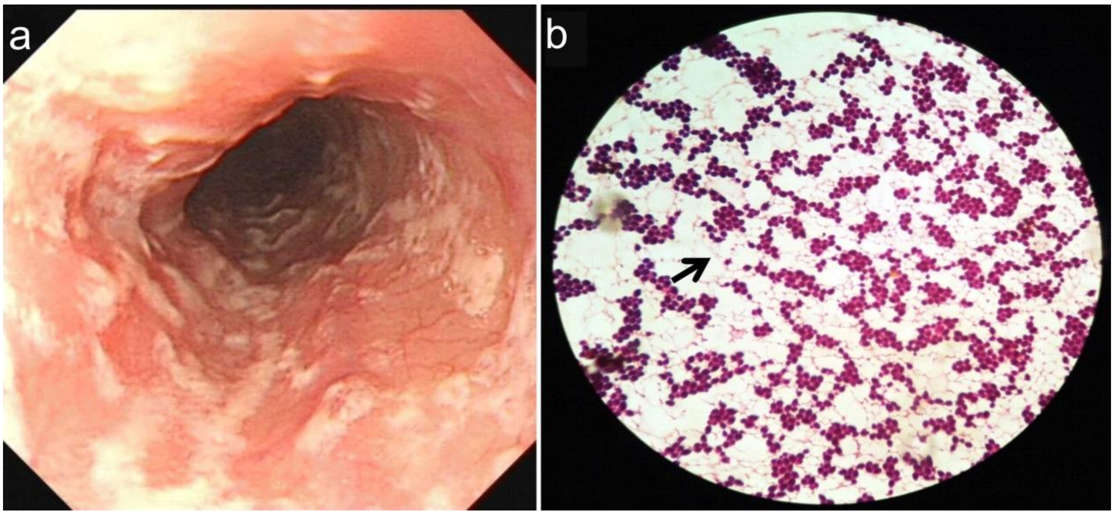

Supplementary Figure S2 *Candida* oesophagitis detected by endoscopy in this study

a *Candida* oesophagitis (Endoscope); **b** *Candida* spores (Microscope×400, Gram stain), the black arrow points to the spores.

Supplementary Table S1 Clinical scores comparison between two groups

**Scores**	**Eso-N (n=33)**	**Eso (n=33)**	***p* value**
**APACHE II**			
Day 4	6.00±3.86	4.78±2.13	0.197
Day 7	2.24±1.84	2.44±1.80	0.722
**SIRS**			
Day 4	1.90±1.68	2.04±1.19	0.700
Day 7	0.71±0.90	1.09±0.95	0.591
**Marshall**			
Day 4	1.86±1.01	1.87±0.91	0.966
Day 7	0.48±0. 87	0.65±0.98	0.535

APACHE II, acute physiology and chronic health evaluation; SIRS, systemic inflammatory syndrome.
